# Mass spectrometry-based proteomics data from thousands of HeLa control samples

**DOI:** 10.1038/s41597-024-02922-z

**Published:** 2024-01-23

**Authors:** Henry Webel, Yasset Perez-Riverol, Annelaura Bach Nielsen, Simon Rasmussen

**Affiliations:** 1 Novo Nordisk Foundation Center for Protein Research, Faculty of Health and Medical Sciences, University of Copenhagen, Copenhagen N, Denmark; 2 European Molecular Biology Laboratory, European Bioinformatics Institute (EMBL-EBI), Wellcome Trust Genome Campus, Hinxton, Cambridge, CB10 1SD UK; 3 The Novo Nordisk Foundation Center for Genomic Mechanisms of Disease, Broad Institute of MIT and Harvard, Cambridge, MA 02142 USA

**Keywords:** Data publication and archiving, Machine learning, Data integration

## Abstract

Here we provide a curated, large scale, label free mass spectrometry-based proteomics data set derived from HeLa cell lines for general purpose machine learning and analysis. Data access and filtering is a tedious task, which takes up considerable amounts of time for researchers. Therefore we provide machine based metadata for easy selection and overview along the 7,444 raw files and MaxQuant search output. For convenience, we provide three filtered and aggregated development datasets on the protein groups, peptides and precursors level. Next to providing easy to access training data, we provide a SDRF file annotating each raw file with instrument settings allowing automated reprocessing. We encourage others to enlarge this data set by instrument runs of further HeLa samples from different machine types by providing our workflows and analysis scripts.

## Background & Summary

Mass spectrometry-based (MS-based) proteomics aims to identify and quantify the complex proteome of a wide variety of biological samples in a reproducible and precise way. MS-based proteomics studies of clinical tissues and blood samples can be used to investigate the molecular disease mechanisms on the level of the proteome, which is the set of proteins in an organism used in molecular pathways and the functioning of a cell in general^1,2^.

The data presented here was collected purely for quality control (QC) and maintenance (MNT) purposes, without the initial intent to use it any further. QC samples were run during large clinical cohort measurements to repeatedly check MS instrument performance and MNT runs are produced after cleaning or in regular intervals for the same purpose. In MS-based proteomics, especially in data-dependent acquisition techniques, technical noise leads to noise in the identified and quantified proteome. Measuring repeatedly a similar biological replicate of HeLa cell lines allowed us to study the performance of machine learning models in the absence of experiment-induced biological variation. We hypothesized that this data could be used to explore self-supervised deep learning models for imputing MS-based proteomics data on the basis of ionised peptides generated from denatured proteins^3^. Therefore in a companion paper we adapted three deep learning architectures for imputation of proteomics data, defined a workflow for model comparison and analysed the effect of imputation using different methods on a downstream analysis task.

**Fig. 1:**
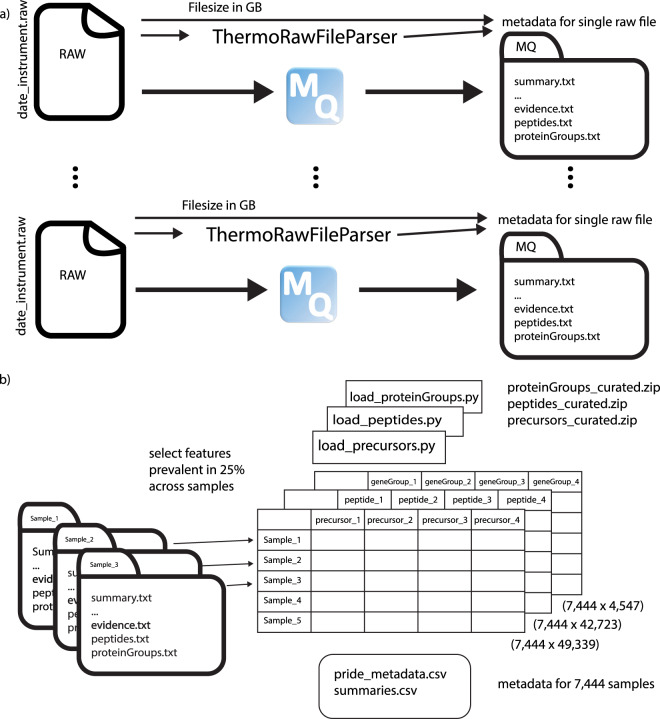
Overview of data collection and aggregated data. (**a**) Each raw file’s size and metadata is recorded. Adding the *summary.txt* information, these form the information collected in ***pride_metadata.csv***. Additionally, summaries of the MQ search are provided in ***summaries.csv***. (**b**) The *summary.txt*, *evidence.txt*, *peptides.txt*, *proteinGroups.txt* and metadata are provided as curated aggregated datasets next to code snippets in python to load the data.

In order to have relative homogenous development datasets, we collected QC and MNT measurements of HeLa cell lines from two collaborating laboratories. The HeLa measurements are based on two cell line strains. In 2018 one of them was selected to consolidate the two laboratories and all measurements were after a short transition phase taken with only one of them. As it is known that there is continued differentiation in HeLa cell lines the underlying biological sample is not expected to be a pure technical replicate^4^. We provide the documented metadata from raw machine files indicating the exact machine type in ***pride_metadata.csv***. We outlined data collection in Fig. 1. In order to allow easier reprocessing of the data, we added a Sample and Data Relationship Format (SDRF) file which allows automated reprocessing of samples^5^.

In summary, this work offers HeLa measurements of MNT and QC runs over a long time window, which is an otherwise unused data pool. We provide functionality to collect a large number of runs in the form of Snakemake workflows next to easy to use aggregated dumps as datasets for tool development. Furthermore, we encourage sharing of more HeLa MNT and QC measurements to increase the pool of data.

We envision that such data can be used to study fluctuations in the measurement of certain machine types, denoising of samples, the development of algorithms that aggregate low level (e.g, precursors) to higher level identifications (e.g., protein groups) or to test new or reference workflows^6,7^.

## Methods

### Process for selecting 7,444 measurement runs

The HeLa cell lines were repeatedly measured as MNT and QC of the mass spectrometers at Novo Nordisk Foundation Center for Protein Research (Copenhagen, Denmark) and Max Planck Institute of Biochemistry (Martinsried, Germany). MNT samples were run after instrument cleaning or repair and QC samples were run during the measurement of larger cohorts to monitor instrument performance over time using HeLa as a reference sample. The standard protocol reduces all alkylates and cysteines^8^. As this data is composed over many years, it contains acquisitions using many machines, various liquid chromatography methods, column lengths and injection methods. The machine identifier is stored in the raw data, however most information w.r.t. chromatography was not recorded. The most widely used instrument for providing gradients for chromatographic separation was a Thermo EASY-nLC 1200, although EVOSEP ONE was used for some runs. In total 50,521 raw files were collected, including mostly single-shot DDA runs of samples. However, there were also a few DIA or fractionated measurements available.

Of the 50,521 raw-files, we were able to process 11,062 DDA runs one by one with MaxQuant 1.6.12^9^ yielding between 0 to 54,316 peptides identified. We then selected runs with at least 15,000 identified peptides based on the information in the *summary.txt*, which gave us 7,484 runs with a minimum raw file size of 0.618 GB. We removed duplicated runs using the creation date and machine identifier and finally had a total of 7,444 unique runs which were uploaded to PRIDE. We extracted metadata information using ThermoFisherRawFileParser and provided this for the selected 7,444 samples.

### Sample preparation protocol

The cells were lysed by different labs and researchers, so the exact procedure might differ slightly over time from the reference protocol uploaded to a protocol preprint server^8^. Although on a per sample basis the exact protocol could not be recovered, all proteins are expected to be digested using trypsin. The injection volume ranges from one to seven microliter.

### Processing steps of a single raw file

We processed raw files using a Snakemake^10^ workflow as a single run in MaxQuant 1.6.12 (ref. ^9^) yielding single abundances for precursor, aggregated peptide and protein group intensities using LFQ. For the DDA searches with MaxQuant we used a UNIPROT human reference proteome database 2019_05 release, containing 20,950 canonical and 75,468 additional sequences. Contaminants were controlled using the default contaminants fasta shipped with MaxQuant. In total three fasta files were uploaded containing the canonical (**UP000005640_9606.fasta**), the additional (**UP000005640_9606_additional.fasta**) and the contaminant (**contaminants.fasta**) sequences. For each individual MaxQuant search, i.e. without match-between-runs, we only specified *Oxidation (M)* and *Acetyl (Protein N-term)* as variable modifications, a maximum of two missed cleavages, at least 7 amino acids per peptide, zero unique peptides, a MS1 first search tolerance and MS2 tolerance of 20 ppm, a MS1 main search main tolerance of 4.5 ppm, and a peptide spectrum match and protein false discovery rate of 1 percent. All parameter files were parsed and uploaded as ***maxquant_parameters.csv***, although besides the number of threads MaxQuant can use, all parameters were identical. From the MaxQuant search of each file, we dumped the tab separated files to PRIDE along the associated raw file. In a companion paper we used the “evidence.txt” for precursor quantifications, “peptides.txt” for aggregated peptides and “proteinGroups.txt” for protein groups referencing them by their gene group^3^.

### Ready to use development dataset

We created a curated, ready-to-use development dataset for three levels of the data for convenience: protein groups, peptides and precursors. The filtered aggregation is provided as zipped folders for these three levels. In brief, filtering was done using a feature completeness cutoff of 25 percent, i.e. that a feature had to be observed in at least 25% of the 7,444 samples. Zero or missing intensity entries were discarded and depending on the file type certain filtering was performed. This is extensively described in Webel *et. al*^3^. First, for protein groups, i.e. proteinGroups.txt files, we dropped “Only_identified_by_site”, “Reverse” and “Potential_contaminent” entries. Then we dropped entries without a “Gene name” and used “Gene name” as identifiers and selected entries with a maximum “Intensity” if one gene set was used for more than one protein group. Second, for aggregated peptides, i.e. peptides.txt files, we used the “Intensity” column for LFQ intensities and used “Sequence” as unique identifiers. Last, for precursors, i.e. evidence.txt files, we dropped potential contaminant entries, zero intensity entries as they provided no quantification for an identified feature, used the “Intensity” column for the label-free quantitation intensities, used “Sequence” and “Charge” as identifiers, and finally selected the entry with maximum intensity for non unique combinations of “Sequence” and “Charge” as this normally corresponds to the best Andromeda score. We did this as we were not interested in modifications and therefore neglected the number of available modified peptides.

## Data Records

The data is available at PRIDE PXD042233^11^. Each uploaded raw file has a MaxQuant search output associated with a set of standard text files as described on their website. Each run was identified by its recording date and the machine identifier as stored in the raw file. The entire MaxQuant folder containing text file based output was stored as a zipped folder with the same identifier as the raw file. These files contained the three data levels which were used in our original publication^3^ as well as e.g. the *msms.txt* and *allPeptides.txt files*. First, the *proteinGroups.txt* file contained all proteinGroups found by searching the associated raw file. Second, the *peptides.txt* file contains aggregated evidence for each peptide identified. Third, the *evidence.txt* file contained all aggregated evidence for each precursor, i.e. peptide uniquely identified by its charge and modification. The data was then further filtered, e.g. by removing contaminants, reversed, non quantified or rare identified sequences, meaning precursors, peptides or protein groups.

**Fig. 2:**
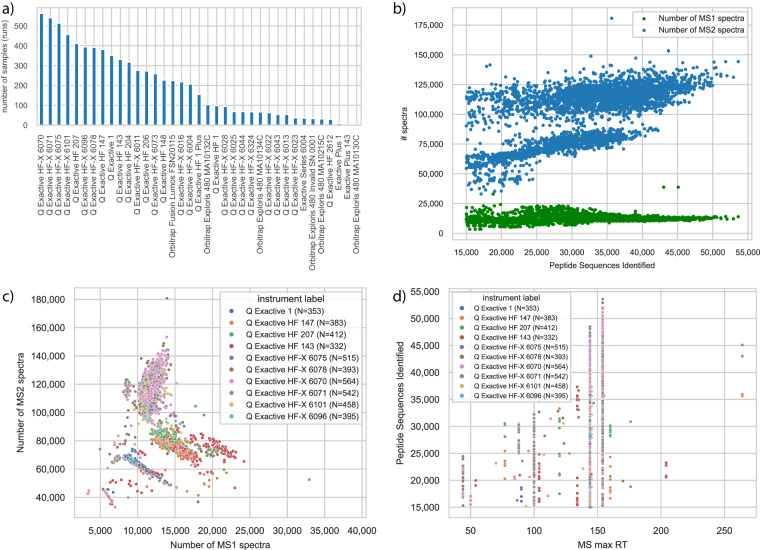
Overview of samples (MS runs) of HeLa. **(a)** Number of samples for each of 37 instruments. See Table 1 for exact numbers. **(b)** MS1 and MS2 spectra in sample over number of identified spectra. Plots are based on ***pride_metadata.csv***. **(c)** Number of MS1 spectra vs MS2 spectra in sample for ten instruments with most measurements. **(d)** Identified spectra over maximum retention time (RT) of sample.

The aggregated and filtered data of all runs ***geneGroups_aggregated.zip***, ***peptides_aggregated.zip*** and ***precursors_aggregated.zip*** contain the data with features in columns and samples in rows, as well as transposed which is the more classical used view in proteomics: samples in columns and features in rows. Note that feature here corresponds to the output of MaxQuant on the evidence, i.e. precursor level, the peptides level and the protein group level identified by gene group. Each zip file contains a brief python script indicating how to load the data into a pandas DataFrame^12^. For further processing it is highly recommended to save the desired selection in a binary format for faster loading. Selected metadata is provided in a separate csv file, called ***pride_metadata.csv***. It combines ThermoRawFileParser^13^ metadata from the raw files with the size of the raw files as well as the summary information of the MaxQuant run. This file can be used to select data of interest, e.g. by selecting files associated with certain instruments, see Table 1 and Fig. 2a. The aggregated summary information from MaxQuant is provided additionally separately in ***mq_summaries.csv***. Based on this information users might select samples according to their assessment of quality from the provided runs with varying quality in terms of identified peptide sequences.

**Table 1:** Instrument information from ThermoRawFileParser and associated counts in 7,444 raw files.

Instrument label	Instrument label	Count
Q Exactive Plus Orbitrap	Q-Exactive-Plus-Orbitrap_1	9
	Q-Exactive-Plus-Orbitrap_143	3
Q Exactive Orbitrap	Q-Exactive-Orbitrap_1	353
Q Exactive HF-X Orbitrap	Q-Exactive-HF-X-Orbitrap_6070	564
	Q-Exactive-HF-X-Orbitrap_6071	542
	Q-Exactive-HF-X-Orbitrap_6075	515
	Q-Exactive-HF-X-Orbitrap_6101	458
	Q-Exactive-HF-X-Orbitrap_6096	395
	Q-Exactive-HF-X-Orbitrap_6078	393
	Q-Exactive-HF-X-Orbitrap_6011	277
	Q-Exactive-HF-X-Orbitrap_6073	260
	Q-Exactive-HF-X-Orbitrap_6016	219
	Q-Exactive-HF-X-Orbitrap_6004	208
	Q-Exactive-HF-X-Orbitrap_6028	95
	Q-Exactive-HF-X-Orbitrap_6025	69
	Q-Exactive-HF-X-Orbitrap_6044	69
	Q-Exactive-HF-X-Orbitrap_6324	68
	Q-Exactive-HF-X-Orbitrap_6022	64
	Q-Exactive-HF-X-Orbitrap_6013	55
	Q-Exactive-HF-X-Orbitrap_6043	55
	Q-Exactive-HF-X-Orbitrap_6023	38
Q Exactive HF Orbitrap	Q-Exactive-HF-Orbitrap_207	412
	Q-Exactive-HF-Orbitrap_147	383
	Q-Exactive-HF-Orbitrap_143	332
	Q-Exactive-HF-Orbitrap_204	319
	Q-Exactive-HF-Orbitrap_206	274
	Q-Exactive-HF-Orbitrap_148	228
	Q-Exactive-HF-Orbitrap_1_Plus	156
	Q-Exactive-HF-Orbitrap_1	99
	Q-Exactive-HF-Orbitrap_2612	30
Orbitrap Fusion Lumos	Orbitrap-Fusion-Lumos_FSN20115	226
Orbitrap Exploris Slot #134	Orbitrap-Exploris-480_MA10134C	67
Orbitrap Exploris Slot #130	Orbitrap-Exploris-480_MA10130C	2
Orbitrap Exploris Slot #0215	Orbitrap-Exploris-480_MA10215C	32
Orbitrap Exploris Slot #0132	Orbitrap-Exploris-480_MA10132C	105
MA-MP9	Orbitrap-Exploris-480_Invalid_SN_0001	34
Exactive Series Orbitrap	Exactive-Series-Orbitrap_6004	36

A set of curated single run dumps for easy user-defined aggregation is provided for the three data levels: ***proteinGroups_single_dumps.zip***, ***peptides_single_dumps.zip*** and ***precursors_single_dumps.zip*** contain each run filtered for a subset of columns and a subset of the entries which are not associated with contaminants or reversed sequences (see above). The id mappings as given by MaxQuant between the three data levels are also provided, i.e. *Evidence IDs*, *Peptide IDs* and *Protein groups IDs*. The set of curated single run dumps can be used to aggregate data with a lower threshold than in the aggregated version - potentially for a subset of the runs.

The mass spectrometry proteomics data and curated dumps have been deposited to the ProteomeXchange Consortium via the PRIDE^14^ partner repository with the dataset identifier PXD042233.

## Technical Validation

We validated that the metadata associated to a raw file is unique in the uploaded dataset based on its creation date and machine identifier. Initially, some files were duplicated and reprocessed with differing file names. Using the information from the summary.txt of the MaxQuant output, we filtered runs by including only runs with a minimum of 15,000 identified peptides. The samples can be further selected using their ratio of identified to MS1 or MS2 spectra (Fig. 2b), their ratio MS1 to MS2 spectra recorded (Fig. 2c), their retention time (Fig. 2d).

**Fig. 3:**
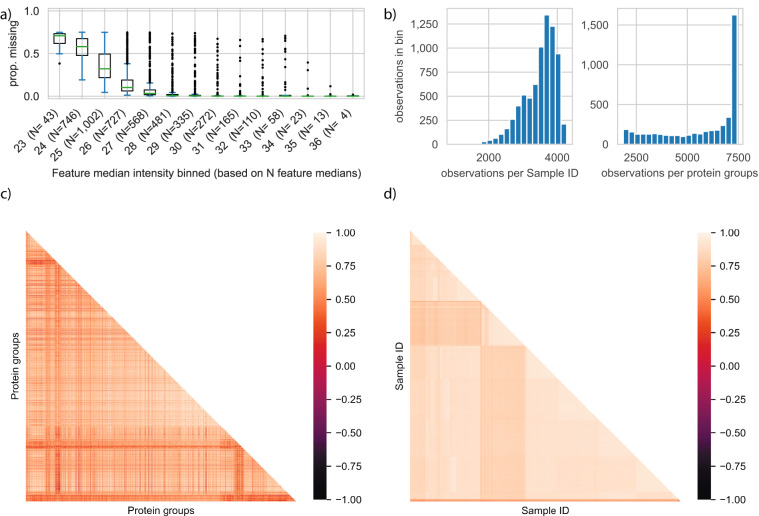
Protein groups analysis for 7,444 samples. **(a)** Proportion of missing values for protein groups which are grouped by their median intensity in bins of integer values. The number of features in a bin is given in parentheses. **(b)** Number of protein groups per sample binned by total of protein groups in sample (left) and number of samples per protein groups binned by total of samples (right). **(c)** Correlation plot between protein groups ordered by missing value pattern clustering of protein groups. **(d)** Correlation plot between samples ordered by missing value pattern clustering of samples.

**Fig. 4:**
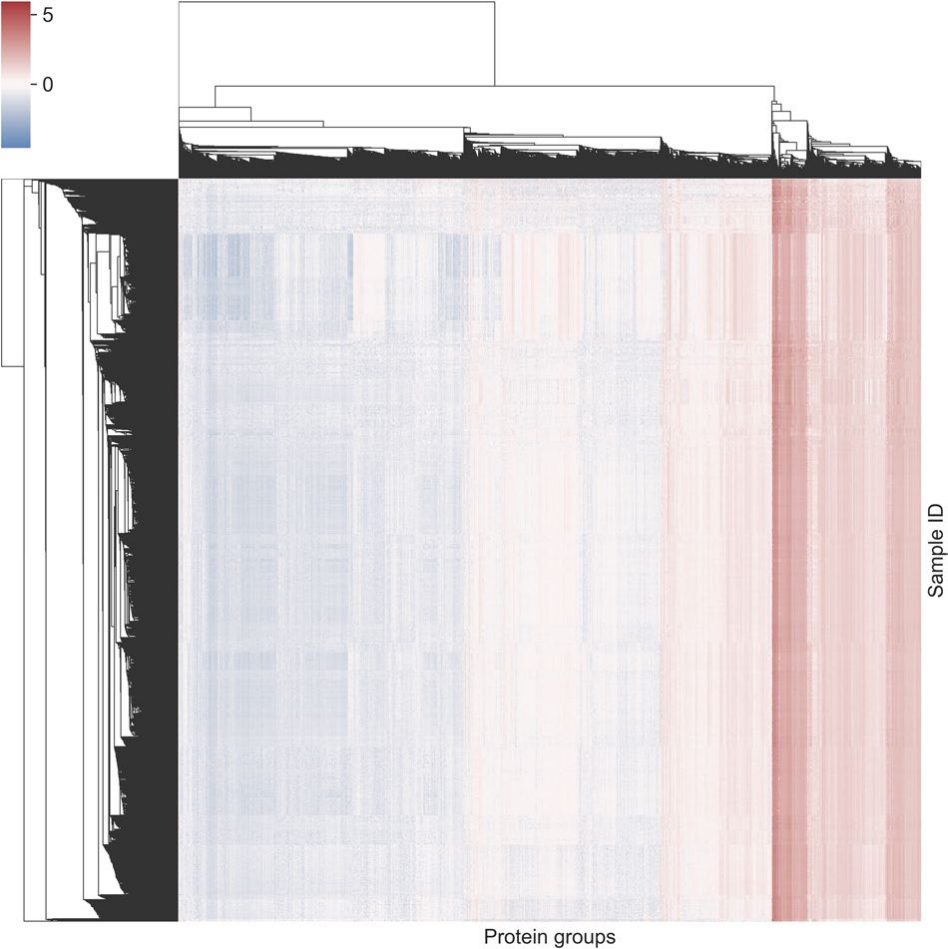
Hierarchical clustering of protein groups and samples using z-score normalised intensities. On the x-axis protein groups and on the y-axis samples are grouped using their correlations across samples or protein groups. Blue indicates relative low abundant protein groups and red relative high abundant protein groups for standard normalised samples.

Based on the clustering of the missing value pattern, we could observe that more low abundant protein groups have a higher percentage of missing values, as expected (Fig. 3a). Most samples had more than 3,000 protein groups quantified, and over 1,500 protein groups were present in nearly all the samples (Fig. 3b). Pearson correlations between samples and most protein groups were positively correlated to each other (Fig. 3c,d). Clustering of samples and protein groups using Pearson correlation on normalised intensities indicated that protein groups clustered by their normalised intensities and samples form groups visually (Fig. 4). This means that large clusters of the data provide a relatively homogenous intensity distribution between samples.

## Usage Notes

The curated data dumps ***geneGroups_aggregated.zip, peptides_aggregated.zip*** and ***precursors_aggregated.zip*** are ready to use and each contains a brief python script showing how to load the data into memory using python. We recommend selecting the data of interest using the provided metadata in ***pride_metadata.csv*** and store the processed data in a binary output format for improved file reading speed. The protein groups, peptides and precursors dataset are provided in wide format, dumped both with the samples in the rows and features in the columns, and reversed. File loading using pandas^12^ in python is provided by a small script contained in the zipped folders for each level. See github.com/RasmussenLab/hela_qc_mnt_data for the latest instructions.

Using the original MaxQuant zipped output folders, it is required to download, unzip and then read in the tab separated files, which have a ‘txt’ file extension instead of ‘tsv’, for further processing.

### Searching samples in a distributed way

A snakemake workflow^10^ for processing of the uploaded HeLa samples is available. It can be used as a skeleton to re-analyze the uploaded Hela samples with a different search engine than MaxQuant, or your own files. It is based on the assumptions that files are located at a remote server and each file is processed individually.

### Reading metadata for raw files

A snakemake workflow^10^ for reading the file metadata of the Thermo Fisher instruments is provided. It downloads the data from a FTP server (or finds the files locally) and then uses the ThermoRawFileParser^13^ to extract the metadata from each file.

## Data Availability

The code used for preparing the PRIDE data upload, the creation of curated data views on the University of Copenhagen FTP large file storage called ERDA, the workflows for sample raw file processing are available on github.com/RasmussenLab/hela_qc_mnt_data. The software used for processing is provided as a python package in the provided GitHub repository.
